# Experiences of discrimination and self-reported health in Chinese migrants: a structural equation model

**DOI:** 10.1186/s12889-020-09588-3

**Published:** 2020-09-29

**Authors:** Lian Tong, Ichiro Kawachi

**Affiliations:** 1 Department of Maternal and Child Health, School of Public Health, Fudan University, Shanghai, China; 2 Department of Social and Behavioral Sciences, Harvard T.H. Chan School of Public Health, Boston, USA; 3 Key Laboratory Public Health Safety, Chinese Ministry of Education, P.O. Box 244, 138 Yixueyuan Road, Shanghai, 200032 China

**Keywords:** Migrants, Discrimination, Social integration, Stress, Health

## Abstract

**Background:**

Many migrants suffer from discrimination and poor health in China. We sought to examine the associations between experiences of discrimination and self-reported health among internal migrants in China, as well as the mediators of social integration and perceived stress.

**Methods:**

The data was obtained from a specific survey of migrants, as a part of the National Health and Family Planning Dynamic Monitoring for Migrants conducted in 2014. A total of 15,999 migrants aged 15 to 59 years were recruited by a stratified, multistage clustered sampling procedure in eight Chinese cities. Structural Equation Modeling (SEM) was conducted.

**Results:**

The results indicated that experiences of discrimination were associated with worse self-reported health (β = − 0.32, *P* < 0.001), less social integration (β = − 0.25, *P* < 0.001), as well as higher perceived stress (β = 0.21, *P* < 0.01). Both objectively measured socioeconomic status (β = 0.21, *P* < 0.001) and subjective social status (β = 0.21, *P* < 0.01) had significantly positive correlations with self-reported health.

**Conclusions:**

The discrimination, social exclusion and perceived stress experienced by migrants have significant implications on their health.

## Background

With rapid urbanization, the Chinese population is currently witnessing an unprecedented level of mass internal migration. According to a Migrant Population Development report, China’s internal migrant population had reached 245 million in 2016, comprising more than a sixth of the nation’ s total population [1]. The nature of such intercity migration involve migrants from both rural areas (rural migrants) and from other urban areas (urban migrants). While internal migrants endow an indispensable contribution towards rapid economic development in China, they remain socially and economically disadvantaged in various domains of life [2]. Numerous studies have revealed that Chinese internal migrants are at elevated risk for physical and mental health problems [3, 4]. Several possible factors contributing to migrants’ debilitating health have been proposed, including experiences of discrimination, acculturative stress, and economic hardships [5]. However, the underlying mechanism behind the association of internal migration and possible deteriorations in health remains unclear.

Due to significant disparities in culture, economic development, and social environments across Chinese regions, a large number of migrants, especially rural-to-urban workers experience widespread social inclusion problems and both institutional and interpersonal discrimination [5]. Discrimination towards migrants can occur through variegated sectors, including their daily life, within the labor market, and public service [6]. There is general evidence suggesting a correlation between experiences of discrimination and reduced self-perceived health [7]. A comprehensive review by Pascoe & Richman (2009) showed the harmful health effects of discrimination across a range of mental health, well-being and specific types of physical health problems, including self-reported poor health. The authors highlighted that perceived discrimination may affect health through a range of psychological and physiological stress responses and behaviors [8], which is likely observed in domestic migrants experiencing social segmentation in China [2, 9]. There are currently limited studies showing that mental health among rural-to-urban migrants are negatively influenced by discrimination in daily life and perceived social inequity [9]. Considering the wellbeing of the escalating migrant population in China, more attention on the relationship between discrimination and migrant health outcomes is required. The current study is focusing on the interpersonal discrimination of migrants.

Various theories have shaped our understanding of migrants’ experiences in prejudice and discrimination within the host society, including in relation to health. In this study, we focus on the integration theory and stress theory [10, 11]. Researchers measure migrant integration within the host society through four dimensions: social, economic, cultural, and political [10, 12]. The experience of migrating and assimilating into a host society can be socially and mentally daunting. Unsuccessful attempts towards adaptation may exacerbate the migrants’ perceived stress, poor overall health, and reduce well-being. Social integration, as one of the main dimensions of newcomers’ experiences in the host society was demonstrated to be significantly related to self-reported health of migrants [13]. However, it is unknown whether it would mitigate the association between discrimination and perceived health. Social integration is defined as a process during which newcomers or minorities are incorporated into the social structure of the host society [14]. This process required mutual accommodation and adjustments from immigrants and members of the host society. However, social integration is often complicated by negative attitudes and discrimination. A study investigating the possible role of social integration in mediating the association between interpersonal discrimination and self-reported health of migrants would thus be valuable.

We propose that in addition to social integration, perceived stress by migrants is another underlying pathway that associates with the poor health related to discrimination. The stress theory and empirical studies have suggested that high levels of perceived stress result in reduced health outcomes [15, 16]. The global literature has indicated a negative impact of acculturative stress on both physical and mental health among international migrants [17]. The expedited lifestyle that emphasizes immigrants’ assimilation into life within the host society and acculturative stress contributes to their vulnerability in health [18] (Li, Meng, & Wang, et al., 2017). Besides acculturative stress, migrants are known to suffer from many other types of stress arising from work, family and interpersonal-related difficulties. They are thus more likely to experience higher levels of psychological distress compared to local residents [19, 20].

Economic integration is another key dimension of integration, and plays a critical role in the development of migrants’ social condition. Convergence appears to be the most important step in the process of economic integration [14], and a lack of entry into the economic mainstream would make other forms of integration difficult [14]. Furthermore, experimental findings on income inequality suggest that it is not the absolute socioeconomic resources, but rather the perception of inequality accompanied by “psychological pain” that affects health [21]. Social income inequality is negatively correlated with the health status of migrants [13].

Social status is an important predictor for a wide range of health outcomes [22]. In particular, the subjective social status (SSS) is a comprehensive measure of one’s social position that is related to several poor health outcomes and risk factors for disease [23]. Similar to objective socio-economic status, subjective perceptions of status are also consistently linked with mental and physical health outcomes of Chinese [24]. It has been indicated that Chinese rural-to-urban migrants evaluate their subjective well-being not only through their financial achievement, but also from their perceptions and beliefs about their relative social status [25]. Thus, it is essential to conduct a study under the consideration of both objective income and subjective social status of internal migrants.

In summary, self-reported health of migrants can be formed in complicated ways during the adaptation process in the host society. However, most studies on health related factors for migrants have merely focused on a single aspect of social life, such as acculturation or economic status [13, 26]. Very limited studies have illustrated health status from social perspectives, e.g. social capital and social support [27, 28]. To the best of our knowledge, no studies have examined the health related factors from a more comprehensive social perspective, which include discrimination, social integration, stress, as well as SSS. The present study will fill these gaps by examining the associations between perceived interpersonal discrimination and self-reported health, in the context of social integration and perceived stress. The objective and subjective socio-economic status of migrants is concurrently considered.

## Methods

### Participants

The data utilized in this study was obtained from a survey of National Health and Family Planning Dynamic Monitoring for Migrant Workers, by the National Health and Family Planning Commission of China in May 2014. Migrant workers are defined as people whose *hukou* (household registration) is not based in the city where they lived for more than a month at the time of the survey. The survey targeted eight cities or districts located in eight different provinces in China (e. g. Chaoyang district in Beijing, Jiaxing city in Zhejiang province). In each city/district, 2000 households of migrant workers were recruited by stratified, multistage clustered Probability and Proportionate to Size (PPS) sampling. Resident committees in each unit of City Street and Village were selected from the qualified cities or urban districts by the method of PPS. All the migrated families administrated by the selected city street or rural village committees were divided into survey groups. Then, 100 qualified migrant families were selected. Finally, 20 migrants were selected depending on their gender, age and the time of migration. If the selected migrants were not available during the investigation, they will be replaced by other individuals according to the principle of “same gender, similar age and a similar migrated time in the host city”.

In each household, only one person aged 15 to 59 years was selected. A total of 15,999 migrants were included in our final study, with 55% being male. The mean age of the migrants is 32.6 years old (SD = 8.7), with men being 32.9 years old (SD = 8.9), and women being 32.1 years old (SD = 8.5). 86% of the migrant workers hold an agriculture *hukou*. 60% of the migrants completed middle school as their highest level of education. Over half of the migrant workers migrated across from other provinces (54.8%) and 69.4% stayed in a city for over 5 years (see Table 1).

**Table 1:** The differences of self-reported health among migrant workers in socio-demographic characteristics

	N	%	Self-report health $\overline{\mathrm{x}}$ (SD)	Z / *χ*^2^	*P*
Gender					
Male	8799	55.0	23.4 (3.8)	-8.03	<.0001
Female	7200	45.0	23.0 (3.9)		
Age					
< 18	291	1.8	24.3 (3.5)	151.64	<.0001
18–29	6070	37.9	23.6 (3.7)		
29–39	5668	35.4	23.1 (3.9)		
39–49	3349	20.9	22.8 (4.0)		
≥ 49	621	3.9	22.3 (4.1)		
Marital status				64.28	<.0001
Unmarried	4056	(25.35)	23.6 (3.7)		
Married	11,540	(72.13)	23.1 (3.9)		
Remarried	169	(1.06)	22.8 (4.0)		
Divorced	198	(1.24)	23.2 (4.0)		
Widowed	36	(0.23)	21.0 (4.5)		
Hukou					
Agriculture	13,759	86.0	23.3 (3.9)	-4.75	<.0001
Non-agriculture	2240	14.0	22.9 (3.9)		
Education				50.27	<.0001
Illiteracy or primary school	1505	9.4	22.7 (4.0)		
Middle school	8085	50.5	23.3 (3.8)		
High school	4051	25.3	23.4 (3.8)		
College and above	2358	14.7	22.9 (3.9)		
Personal month net income (USD)					
< 300	2412	15.1	22.9 (4.1)	12.62	0.0055
300–750	10,578	66.1	23.2 (3.8)		
750–1500	2466	15.4	23.3 (3.9)		
≥ 1500	543	3.4	22.9 (4.0)		
Household month income (USD)					
< 300	420	2.6	23.6 (4.1)	12.57	0.0057
300–750	5990	37.4	23.2 (3.9)		
750–1500	7541	47.2	23.1 (3.9)		
≥ 1500	2048	12.8	23.3 (3.8)		
Migrated distance					
Across province	8771	54.8	23.1 (3.9)	3.42	0.0006
With province	7228	45.2	23.3 (3.9)		
Migrant time					
Over five years	11,105	69.4	23.3 (3.8)	−1.95	0.511
Less than five years	4894	30.6	23.1 (3.9)		
Total	15,999	100.0	23.2 (3.7)		

### Ethics approval and consent to participate

The “National Internal Migrant Dynamic Monitoring Survey, 2014” data is publicly available to authorized researchers who have been given permission by the National Population and Family Planning Commission. Written informed consents were obtained from all participants. The analysis of public access data was exempted by the local Institutional Review Board (IRB); as this study involves analyzing existing data anonymously, ethical approval was not required.

### Measures

#### Self-reported health

Self-reported health was assessed by a subscale of general health in the 36-Item Short Form Health Survey (SF-36) [29]. The following items from the SF-36 were used to assess health-related quality of life: (a) In general, would you say your health is …? (b) Compared to 1 year ago, how would you rate your health in general now? (c) I seem to get sick a little easier than other people; (d) I am as healthy as anybody I know; (e) I expect my health to get worse; (f) My health is excellent. Participants were required to rate their perceived health on a five-point Likert scale. The options for the first question ranged from excellent to poor, and for the second question are much better now than 1 year ago, somewhat better, about the same, somewhat worse, and much worse now than 1 year ago. For the last four questions, the five response options are strongly agree, mostly agree, not sure, mostly disagree, and strongly disagree. The score from 1 to 5 was used to code each response option. The option indicated the best health status is coding as 5, the option indicated the worst health status is coding as 1. The 6 items are then summed to derive an overall score, with a higher score indicating better self-reported health. The internal consistency (Cronbach’s alpha) of general health was .89.

#### Discrimination

The perceived interpersonal discrimination rating scale was applied in this study, which was demonstrated to be a validated method to assess self-perceived interpersonal discrimination for migrants [30]. The scale was developed based on Everyday Discrimination Scale, which is a proper tool for assessing interpersonal discrimination [31, 32]. The Cronbach’ s alpha in the present sample was acceptable with a value of 0.79. It included the following four items: 1) I think the local-born residents don’t want to see me as one of them; 2) I feel the locals don’t want to be my neighbors; 3) I think the locals don’t like me; 4) I think the locals look down upon me. Participants were asked about their levels of agreement with these statements based on a four-point Likert scale that ranged from 1 to 4: strongly disagree (4), basically disagree (3), basically agree (2), and strongly agree (1). For each item, score of 1 to 4 was coded in the statistic analysis, the option indicated the highest level of discrimination is coding as 4, the option indicated the lowest level of discrimination is coding as 1. The sum of all the scores on the 4 items was tallied for a total score of discrimination. A higher score indicates greater self-perceived discrimination.

#### Social integration

The Social Integration Scale was adopted in this study, which showed good psychometrics characteristics [33]. Eight questions were included in this scale, for example, “I would like to live with locals in the same block (community)”, “I would like to make friend with local people”, “My relatives or myself would like to marry local people”, and “I feel like I belong in this city”. Participants were asked about their levels of agreement with these statements based on a four-point Likert scale: (a) strongly disagree, (b) somewhat disagree, (c) somewhat agree, and (d) strongly agree. The eight items are then summed to derive an overall score of social integration. A higher score indicates better social integration. The Cronbach’s alpha for the social integration scale was 0.92.

#### Perceived stress

The four items of Perceived Stress Scale were selected to evaluate stress levels [34]. The participants were asked to rate their experiences in the past month, which include: a) how frequently do you feel a lack of control over the important things in your life? b) how often do you feel confident in your ability to handle personal problems? c) how often have you felt that things were going your way? d) how often do you feel incapable of coping with all the things that you had to do? Each item was assessed by a five-point Likert scale with 1 for never, 2 for sometimes, 3 for often, 4 for very often, and 5 for always. The first two items are given score reversely when calculating the total score. Then the four items are then summed to derive an overall score. A higher score indicates the greater stress. The internal consistency (Cronbach’s alpha) for the Perceived Stress Scale was .89.

#### Objective socioeconomic status (SES)

Objective SES was indexed by the family monthly income and education level of participants in the present study. Participants were asked to factually report their family monthly income. The family monthly income was chosen as a more sensitive index to reflect their family’s SES than their personal monthly income, because most of the participants were married. We were unable to utilize the occupational classification used in this survey towards the analysis, as it only displays profession types without adequately reflecting the SES of the family.

#### Subjective socioeconomic status (SSS)

The participants’ SSS was assessed by the MacArthur Scale of Subjective Social Status [35, 36]. Participants were first shown a picture of a 10-step ladder and asked to think of it as representing where people stand in China. The scale can be described as follows: The top of the ladder (10th rung) depict people who are the best off – those who have the most money, the highest level of education, and the most respected jobs. At the bottom (1st rung) are the people who are the least affluent – those who have little money, the lowest level of education, and the least respected jobs or are unemployed. Participants were instructed to place themselves in the rung that they felt most represented their relative standing when compared with their social contacts (e.g., friends, family, and work group). This measure was previously used for Chinese rural-to-urban migrants and has been shown to be reliable and valid [37].

### Statistical analysis

The data analysis was conducted by Statistic Analysis System (SAS) version 9.3 (Institute Inc., Cary, NC, USA). Wilcoxon Rank-Sum test for two groups and Kruskal-Wallis test for more than two groups’ comparison were applied to examine the self-report health differences among migrant workers with different socio-demographic characteristics. Structural Equation Modeling (SEM) was performed to clarify the associations among discrimination, social integration, stress and self-report health of migrant workers. The method of SEM is a good method to explore the mediating effect, which is consistent with objectives of this study. SEM is also a good method to explore the causal relationship for cross-sectional data. The model fix indices in our data were good and the model was also consisting with existing finding. In addition, objective and subjective socioeconomic status, gender of migrants (1 for male, 2 for female) and the continuous variable of age were put in the SEM model step by step. To test whether discrimination was significantly related to self-reported health through the potential mediators of social integration and perceived stress, we calculated the Sobel’s z statistic, a commonly used statistic for testing the significance of mediation effect [38].

## Results

Male migrant workers reported better health status than female workers (Z = -8.03, *P* < 0.0001). Older migrant workers had worse self-reported health than younger ones (*χ*^2^ = 151.64, *P* < 0.0001). The self-reported health of migrant workers is also various by their marital status, educational level and monthly income. In addition, migrants migrated across provinces had worse self-reported health than those moved within the province (Z = -3.42, *P* = 0.0006). Migrants with agriculture *hukou* showed worse perceived health status than their counterparts with non-agriculture (Z = -4.75, *P* < .0001) (see Table 1). Partial correlation analysis (see Table 2) showed that self-reported health had a significantly positive correlation with social integration (r = 0.22, *P* < 0.0001), as well as subjective SES (r = 0.22, *P* < 0.0001). Self-reported health had a significant, inverse correlation with discrimination (r = − 0.21, *P* < 0.0001) and perceived stress (r = − 0.37, *P* < 0.0001).

**Table 2:** The partial correlations among discrimination, social integration, stress, SES and self-report health

	Self-report health	Social integration	Discrimination	Perceived stress	Family income
Social integration	0.22***				
Discrimination	−0.21***	−0.51***			
Perceived stress	−0.37***	−0.21***	0.20***		
Family income	0.02*	0.02**	−0.02	−0.05***	
Subjective social status	0.22***	0.18***	−0.15***	−0.25***	0.13***

* *P* < 0.05, ** *P* < 0.01, ***P < 0.0001. Controlled migrants’ age, gender and *hukou*

**Fig. 1:**
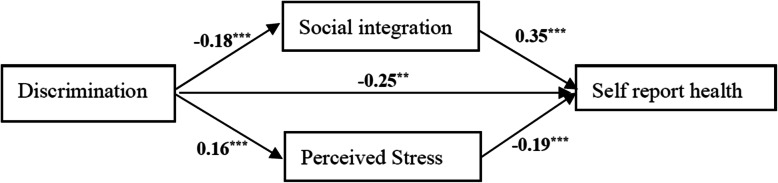
SEM model 1 testing correlations among discrimination, social integration, stress, and self-report health in migrant workers. ** P < 0.01, *** P < 0.001

**Fig. 2:**
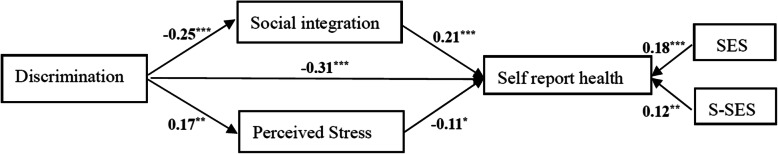
SEM model 2 testing correlations among discrimination, social integration, stress, SES and self-report health in migrant workers. * P < 0.05**, P < 0.01, *** P < 0.001

An analysis with SEM demonstrated that experiences of discrimination had a direct negative impact on the social integration of migrant workers (β = − 0.25, *P* < 0.01), and an indirect negative effect on self-reported health via social integration and perceived stress (see Fig. 1). Sobel z tests for the mediation effects suggested that social integration (Sobel’s z = 5.68, *P* < 0.001) and perceived stress (Sobel’s z = 4.43, *P* < 0.001) significantly mediated the association between discrimination and self-reported health. Social integration also directly affected the self-reported health of migrant workers (β = 0.35, *P* < 0.001). In addition, discrimination was significantly associated with perceived stress (β = 016, *P* < 0.01); and perceived stress in turn linked to self-reported health (β = − 0.19, *P* < 0.001). Family SES were added to the SEM model as well, and it showed that both higher objective SES (β = 0.18, *P* < 0.01) and subjective SES (β = 0.12, *P* < 0.01) significantly associated with better self-report health of migrant workers (see Fig. 2).

**Fig. 3:**
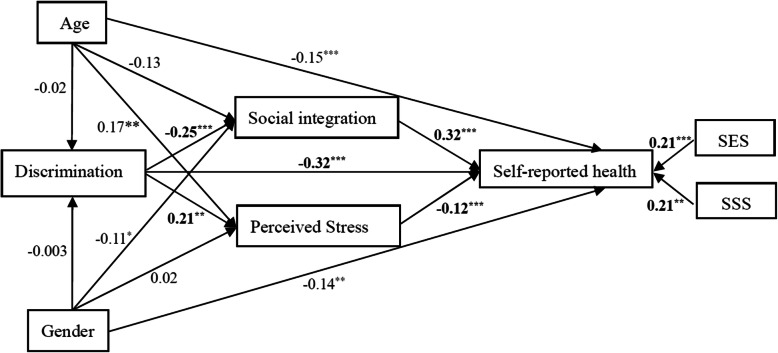
SEM model testing correlations among age, gender, discrimination, social integration, stress, SES and self-report health in migrant workers. * P < 0.05**, P < 0.01, *** P < 0.001。

We simultaneously considered both the gender and age of migrant workers in the SEM model (see Fig. 3). Older migrant workers were found to have worse self-reported health (β = − 0.15, *P* < 0.001), and higher perceived stress (β = 0.17, *P* < 0.01) than young migrant workers. It was also established that women had worse self-reported health than men (β = − 0.14, *P* < 0.01), as well as lower social integration (β = − 0.11, *P* < 0.01). After adjusted for age, gender and SES/SSS of migrant workers, the association between discrimination and self-reported health was still remained (β = − 0.32, *P* < 0.0001). The indices for the goodness of fit for SEM model are moderate and gradually improved (see Table 3). For Model 3, the Comparative Fit Index (CFI) is 0.87, and the Goodness of Fit Index (GFI) is 0.88. The Root Mean Square Error of Approximation (RMSEA) reaches 0.09.

**Table 3:** The goodness of fit for SEM models

	Chi-Square	*P* value of Chi-Square	Comparative Fit Index (CFI)	Goodness of Fit Index (GFI)	Root Mean Square Error of Approximation (RMSEA)	90% Confidence Limit of RMSEA
Model 1	68.44	< 0.001	0.809	0.825	0.104	0.087–0.116
Model 2	199.31	< 0.001	0.837	0.862	0.095	0.079–0.112
Model 3	244.54	< 0.001	0.874	0.878	0.087	0.078–0.099

## Discussion

The present study demonstrated that the everyday discrimination that Chinese migrants frequently experienced is associated with poor self-reported health, while social integration and perceived stress are factors likely to mediate the association. Specifically, migrants who experienced more discrimination reported lower levels of social integration and higher levels of stress, which together contribute to their poor self-reported health outcomes. Discrimination against marginalized groups is a global psychosocial phenomenon, but retains its local character in a given social, cultural, and economic context. China’s internal migrants mostly consist of farmers and small-town residents, who relocate to coastal and first-tier cities. The widespread discrimination experienced by this population is likely to be generated by the presence of dual urban-rural societies in China, as a result of the unique *hukou* system [39, 40]. The *hukou* system causes inequalities in social status between permanent urban and rural residents, leading to prevalent discrimination against rural-to-urban migrants [41]. Sociologists and economists have confirmed that the current *hukou* system plays an important role in the allocation of economic resources, educational opportunities and health care for migrants [42, 43].

If the *hukou* system is fundamentally causing discrimination against migrants, then social integration may work as a mitigating strategy to reduce between-group inequality. The present study builds upon existing knowledge that social integration is positively associated with the self-reported health of migrants [44]. We found that migrants who experienced discrimination reported lower levels of social integration, which further associated with the poor health outcomes of migrants. As discrimination-led societal exclusion may hinder social integration, this can subsequently weaken other factors possibly linked with health status, including social network and support [45]. Deficiencies in personal social networks are plausibly linked to poorer health status [46]. In addition, low levels of social integration may also contribute to disparities in access to health care, another factor which could be negatively associated with migrants’ health status [47, 48]. It has been confirmed that social exclusion in the host society results in unequal social resource allocation, including towards migrants [49]. Social exclusion occurs beyond a materialistic basis and extends to a spiritual and symbolic level, which refers to the social prejudice and discrimination attained by socially dominant groups [50]. There is currently strong evidence suggesting that social exclusion is negatively associated with migrants’ mental health. In previous reports, limited access to full labor rights and experiences of social stigma, discrimination and inequity were found to be the most significant factors contributing to mental health problems in migrants [3].

Moreover, the current study also found that perceived stress exacerbated the associations between discrimination and self-reported health. Daily experiences of discrimination are known to have significant negative impacts on psychological distress and overall quality of life in Chinese migrants [2]. Discrimination is closely related to societal exclusion, bullying, and devaluation on one’s self-worth [51, 52]. The adverse impacts of discrimination-related stress is especially relevant in migrants, who experience pre-existing disadvantages in economic and social conditions, as well as feelings of inadequacy through the adaptation process in host cities [53, 54]. The juxtaposition of these elements with discrimination may pose a risk for health, and convolute the process of adaptation and integration into the new society [55]. A possible pathway is that discrimination acts as a social stressor that sets into motion a process of physiological responses (e.g., allosteric load, elevated blood pressure, cortisol secretions), which over time will have downstream effects on health. Furthermore, when the stressor is prolonged and chronic, it becomes a pre-disposing factor for poor health outcomes in migrants [56, 57]. A previous study indicated that discrimination against migrants do not seem to decrease regardless of their duration of stay in the urban labor market [5].

Beyond social integration and perceived stress, the degree of economic integration is a major structural contributor to the health status of Chinese migrants [30]. Migrants face manifold challenges to their material circumstances, including employment, working conditions, low wages, and cramped living environments. Subjective social status -- or perception of rank on the social hierarchy -- is an additional psychosocial indicator of health outcomes [58]. Indeed in a previous report, subjective feelings of relative social status were even more closely associated with the health of Chinese migrants than objective socio-economic status [30].

This study found that women have worse self-reported health relative to men. Women are known to be a vulnerable group of the migrant population with inferior health status [59]. Gender disparities in health economics encompass elements of injustice, which stems from the dominant values of a society. This is exacerbated in female migrants who may experience gender discrimination, harassment, and gender-based violence on top of discrimination on the basis of their migrant status [60]. Results from the current study did not show differences in perceived stress in female migrants relative to male migrants. We did however observe lower levels of social integration in women compared to men. This may be a result of the low population employment ratio in female migrants, which is 77.5% compared to 93.9% in male migrants. A notable reason for higher unemployment in women is due to their responsibilities in the care of family and children [61]. Between 2011 and 2016, the proportion of women in China’s ‘floating’ population increased, from 47.7% in 2011 to 48.3% in 2016 [56]. Considering this growing trend in the number of female migrants, more attention should be paid to their health status.

### Limitations

Although SEM is suggested to be a good method to explore the causal relationship for cross-sectional data, the surveillance dataset used in the current study, combined with the cross-section design does not allow exploration of causality among discrimination and health outcomes. This study also applied self-reported health statuses, rather than objective health outcomes.

## Conclusions

Discrimination, social exclusion and perceived stress experienced by migrants have significant health implications. Subjective judgments of relative social status levels were associated with self-perceived health, which implies the need for more reforms to promote the rights and welfare of internal migrants. Social policies which targeted different gender, age and socioeconomic groups will also be conducive to the health of migrants. Overall, the migrants’ health status can benefit from improvements in social, economic, and cultural environments.

## Data Availability

The data are owned by a third party and authors do not have permission to share the data. The data can be accessible by submitting application to National Population and Family Planning Commission of China (www.nhfpc.gov.cn).

## Abbreviations

SEM: Structural Equation Modeling
SSS: Subjective social status
SES: Objective socioeconomic status
PPS: Proportionate to size sampling
SD: Standard deviation
IRB: Institutional Review Board
SF-36: 36-Item Short Form Health Survey.
SAS: Statistic Analysis System
CFI: Comparative Fit Index
GFI: Goodness of Fit Index
RMSEA: Root Mean Square Error of Approximation
